# Update on the RESCUEicp decompressive craniectomy trial

**DOI:** 10.1186/cc9732

**Published:** 2011-03-11

**Authors:** PJ Hutchinson, AG Kolias, I Timofeev, E Corteen, M Czosnyka, DK Menon, JD Pickard, PJ Kirkpatrick

**Affiliations:** 1 Academic Division of Neurosurgery, Addenbrooke's Hospital & University of Cambridge, UK; 2 Neurocritical Care Unit & University Department of Anaesthesia, Addenbrooke's Hospital & University of Cambridge, UK

## Introduction

The fundamental pathophysiological process following head injury is the development and propagation of an escalating cycle of brain swelling, increase in intracranial pressure (ICP), reduction in blood supply and oxygen delivery, energy failure and further swelling, enhancing brain injury and poor outcome. The aim of the RESCUEicp trial (Randomised Evaluation of Surgery with Craniectomy for Uncontrollable Elevation of ICP) is to provide class I evidence as to whether decompressive craniectomy is effective for the management of patients with raised and refractory ICP following traumatic brain injury (TBI).

## Methods

An international multicentre randomised trial comparing decompressive craniectomy with optical medical management (including barbiturate therapy). Inclusion criteria: TBI, age 10 to 65, ICP (> 25 mmHg for 1 to 12 hours, refractory to first-line treatment). Exclusion criteria: treatment with barbiturates pre-randomisation, primary decompression (during evacuation of mass lesion), bilateral fixed and dilated pupils, bleeding diathesis, devastating injury unlikely to survive >24 hours. In this study, patients are managed on ICUs using a standard protocol. The major objective of this protocol is to maintain ICP * <*25 mmHg by applying treatment measures in a number of stages. The total number of patients will be 400 (200 in each arm of the study) for a 15% difference in outcome (power = 80%, *P *= 0.05). The primary outcome measure was extended Glasgow Outcome Score at 6 months.

## Results

Over 280 patients have been recruited to date from more than 40 centres in 17 countries. The follow-up rate at 6 months is 96%. To date, evaluation of the first 182 patients shows equal distribution of characteristics between the two arms. Median age is 33 years and 80% of patients are male. Four percent were hypoxic and 13% hypotensive at initial presentation. Seventy percent had an initial GCS of 3 to 8, 19% a GCS of 9 to 12 and 11% a GCS of 13 to 15.

## Conclusions

Randomising patients with TBI to decompressive craniectomy versus optimal medical management is feasible. Whether this operation is effective remains to be seen. We welcome the participation of more centres.
